# Overcoming challenges in rare disease registry integration using the semantic web - a clinical research perspective

**DOI:** 10.1186/s13023-023-02841-z

**Published:** 2023-08-29

**Authors:** Karl Gisslander, Aladdin J Mohammad, Augusto Vaglio, Mark A Little

**Affiliations:** 1 Department of Clinical Sciences - Rheumatology, Lund University, Lund, SE-221 85 Sweden; 2 Department of Medicine, University of Cambridge, Cambridge, UK; 3 Department of Biomedical, Experimental and Clinical Sciences, University of Florence, Florence, Italy; 4 Nephrology and Dialysis Unit, Meyer Children’s Hospital IRCCS, Florence, Italy; 5 TTMI, Trinity Health Kidney Centre, Dublin, Ireland; 6 ADAPT, Trinity College Dublin, Dublin, Ireland

**Keywords:** Rare disease, Semantic web, FAIRification, Data integration, Vasculitis, Registry

## Abstract

The growing number of disease-specific patient registries for rare diseases has highlighted the need for registry interoperability and data linkage, leading to large-scale rare disease data integration projects using Semantic Web based solutions. These technologies may be difficult to grasp for rare disease experts, leading to limited involvement by domain expertise in the data integration process. Here, we propose a data integration framework starting from the perspective of the clinical researcher, allowing for purposeful rare disease registry integration driven by clinical research questions.

## Main text

Disease-specific patient registries are essential for our increased understanding of rare diseases, but these registries remain fragmented, siloed, and lack data sharing mechanisms. Under the auspices of the European Joint Programme for Rare Diseases (EJP RD) and the European Reference Networks (ERNs), and guided by the FAIR (Findable, Accessible, Interoperable, and Reusable) principles for scientific data management, several initiatives have been undertaken to tackle these challenges using Semantic Web based solutions [1, 2]. The Semantic Web is an initiative to represent and publish data resources on the internet to facilitate data linkage and processing. By the extensive use of ontologies, a uniform vocabulary is created across resources, adding “semantic” meaning to the data. This allows for machine-readability of heterogenous data and is the foundation of the FAIR initiative. However, to achieve a meaningful exchange across existing resources, such as rare-disease registries, there is a need for semantic harmonisation of the data-items to uniform ontology concepts. This transformation of registry data to a uniform vocabulary generates what we call the “semantic layer” of the registries. The FAIRVASC project is one of the initiatives utilising Semantic Web technologies, enabling the federation of knowledge between multiple independent registries concerning the rare disease anti-neutrophil cytoplasmic antibody associated vasculitis (AAV) [3].

Through semantic data representation, integrated access and querying, Semantic Web technologies hold the potential for benchmarking of patient care and personalised medicine in diseases where traditional research suffers from small sample sizes. However, the concept of the Semantic Web and its benefits are often difficult and time-consuming to grasp for rare disease researchers and clinicians, leading to limited involvement from domain expertise in the data integration process. This may lead to the development of a semantic layer not fit for the purpose of clinical research. We propose a data integration framework highlighting the need for clinical researcher involvement based on an iterative approach.

Central to the concept of the Semantic Web are ontologies, such as the Orphanet Rare Disease Ontology (ORDO), the Human Phenotype Ontology (HPO), SNOMED CT, Online Mendelian Inheritance in Man (OMIM) and, the Drug Ontology (DRON) enabling interoperability between resources through a common language [4]. In the siloed registries of today, the same concept may be represented in several ways. A translation step to allow for interoperability between the siloed registries is needed. This is the semantic layer, allowing for mapping of registry data to a shared vocabulary. However, exploring the rich environment of biomedical ontologies where obvious standards are lacking and finding the appropriate ontology terms, thus creating the semantic layer requires domain expertise. In FAIRVASC we tackled this through iterative cycles of data harmonisation, technical implementation, and query development driven by competency questions generated by domain expertise, building on existing methodology for ontology development [5–7].

First, a team of domain experts identify a competency question, a clinical research question to be answered through the semantic layer of the federated registries. This competency question is passed on to a team of registry experts with extensive knowledge of local registry data availability, semantics, and structure. In collaboration this team searches standard or widely adopted ontologies for appropriate concepts matching the registry data needed to answer the competency question. The identified ontology concepts, their matching registry variable names and the competency question are then transferred to a team of data managers or computer scientists for the technical implementation of the semantic layer. This technical implementation team are now tasked with two key objectives. First, they build the semantic layer by representing the objects and relationships of the registry data using the identified ontology concepts. Secondly, they build the query to retrieve the information needed to answer the competency question from the semantic layer of the federated registries. Following this cycle of activity, feedback from the implementation from all teams then informs the next iteration.

This easy-to-follow framework for the development of the semantic layer in rare disease registry integration actively involves the clinical researcher and is assuring data interoperability which is fit for the purpose of clinical research. By employing research question driven semantic layer design, we minimise the exposed data, further strengthening the privacy-preserving mechanisms already in place in a federated approach to registry integration. The result is a semantic layer built for the needs of the clinical researcher as opposed to data integration for the sake of data integration. By proposing this easy-to-follow framework to ensure clinical researcher involvement in data integration projects, we hope to incentivise registry owners to share their data and unlock the full potential of the growing number of rare disease registries.

We, as rare disease researchers, see a growing number of data integration projects within the community and an extended implementation of FAIR. From our experience with the FAIRVASC project, successful rare disease registry integration and FAIRification using the Semantic Web requires extensive involvement from domain expertise, starting from research-driven objectives rather than implementation of data integration for the sake of data integration. We, therefore, here propose a framework for the development of the semantic layer needed for data integration in an iterative approach purposefully starting from the clinical research questions.

## Data Availability

Not applicable.
